# DNA Damage Repair and Current Therapeutic Approaches in Gastric Cancer: A Comprehensive Review

**DOI:** 10.3389/fgene.2022.931866

**Published:** 2022-08-12

**Authors:** Menghui Wang, Chuan Xie

**Affiliations:** Department of Gastroenterology, the First Affiliated Hospital of Nanchang University, Nanchang, China

**Keywords:** DNA damage repair, *Helicobacter pylori*, therapeutic approaches, gastric cancer, PARP inhibitors

## Abstract

DNA in cells is frequently damaged by endogenous and exogenous agents. However, comprehensive mechanisms to combat and repair DNA damage have evolved to ensure genomic stability and integrity. Improper DNA damage repair may result in various diseases, including some types of tumors and autoimmune diseases. Therefore, DNA damage repair mechanisms have been proposed as novel antitumor drug targets. To date, numerous drugs targeting DNA damage mechanisms have been developed. For example, PARP inhibitors that elicit synthetic lethality are widely used in individualized cancer therapies. In this review, we describe the latent DNA damage repair mechanisms in gastric cancer, the types of DNA damage that can contribute to the development of gastric cancer, and new therapeutic approaches for gastric cancer that target DNA damage repair pathways.

## Introduction

According to the 2020 Global Cancer Statistics, gastric cancer was responsible for 1,089,103 new cases in 2020 and approximately 768,793 deaths (corresponding to 1 in every 13 deaths universally) worldwide, making it the fifth most frequently diagnosed cancer and the fourth leading cause of cancer-related death (Hyuna et al., 2021). Gastric cancer is the fourth and seventh leading cause of new cancer cases in males and females, respectively, and the fourth and fifth leading cause of cancer-related death in males and females, respectively (Hyuna et al., 2021). Most new cases occur in developing countries, especially in China (Zhou et al., 2020). The global incidence of gastric cancer is 42.6% and the mortality rate is 45.0% (Ferlay et al., 2014). Data from cancer registries have revealed that gastric cancer is more likely to occur in locations where individuals have unhealthy diets, such as remote rural areas (Hongli et al., 2017).

Hence, novel strategies are urgently being researched and developed to improve the prognosis and survival of patients with gastric cancer. Twenty years ago, some studies demonstrated that DNA damage repair (DDR) mechanisms play a significant role in the tumorigenesis, progression, and treatment validity of gastric cancer (Hoeijmakers, 2001). In recent years, some clinical and preclinical studies have concluded that DDR pathway inhibitors may prolong the survival time of patients (Young et al., 2016).

## Latent DNA Damage Repair Mechanisms

Genome instability leads to mutations in DNA repair genes and is a symbol of cancer evolution and one of the universal mechanisms of tumorigenesis (Tian et al., 2015). DNA damage refers to the physical or chemical changes in the DNA found in cells, affecting the interpretation and transmission of genetic information. Extensive DNA damage may subsequently activate oncogenes or inactivate tumor suppressor genes, such as p53 (Kim et al., 2019a). To repair this DNA damage, cells have developed a remarkable mechanism—DDR pathways. DDR pathways play a crucial role in the development of human cells and can repair different types of DNA damage (single-strand breaks (SSBs), pyrimidine dimers, A–C or A–G or T–C or T–G mismatches, DNA interstrand crosslinks, and double-strand breaks (DSBs)) to maintain genomic stability (Sokolova and Naumann, 2019a; Rahman et al., 2020). This damage can be caused by both exogenous and endogenous factors, such as replication fork stalling, reactive oxygen species (ROS) generation, genetically toxic substances, and ultraviolet rays (Sokolova and Naumann, 2019a).

**FIGURE 1:**
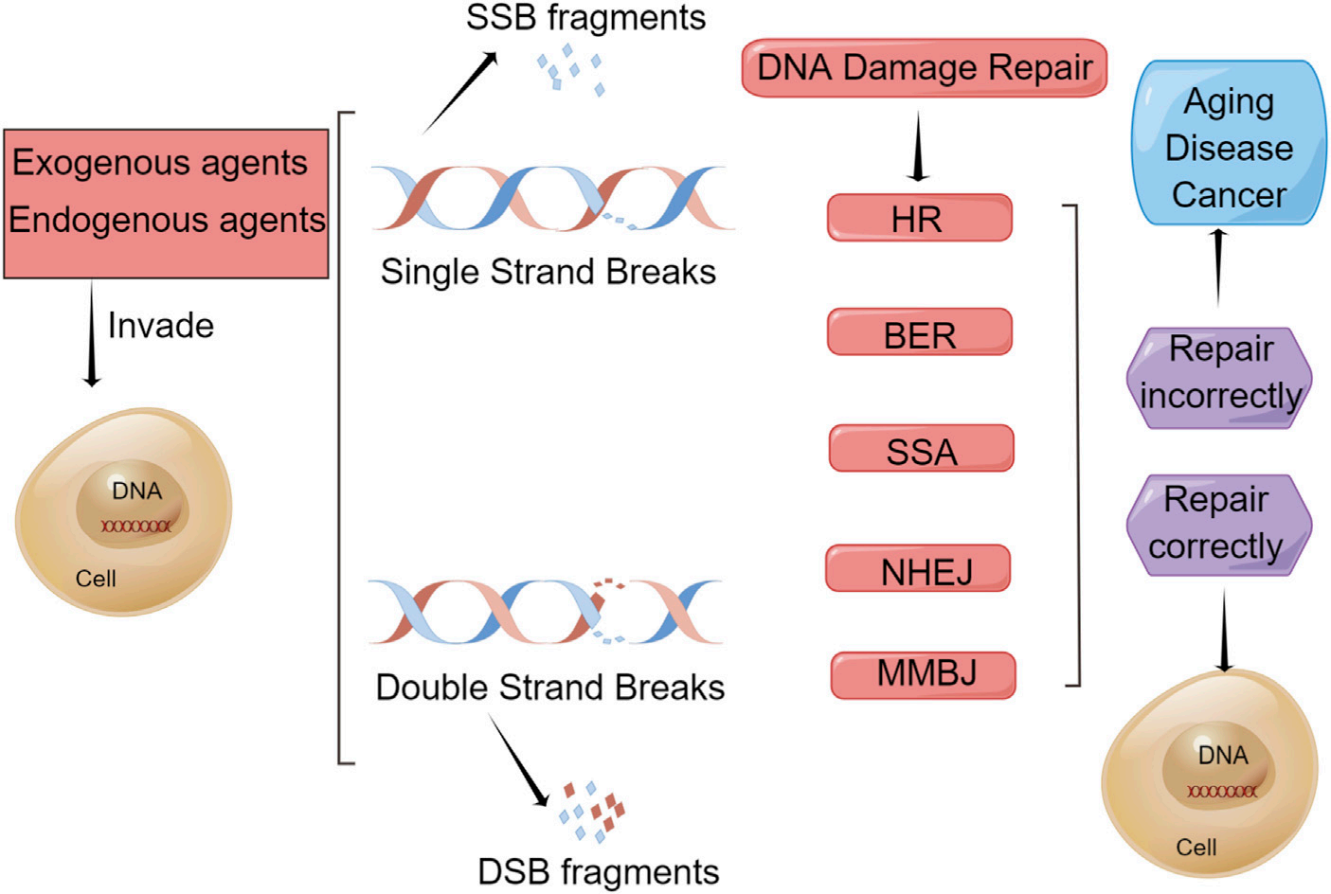
Repair methods and outcomes of DNA damage. Endogenous and exogenous factors can damage DNA in a cell, such as reactive oxygen species (ROS) generation, replication fork stalling, chemical agents, ultraviolet radiation, and ionizing radiation, which cause single-strand breaks (SSBs) and double-strand breaks (DSBs) to produce SSB fragments and DSB fragments, respectively. To mitigate this damage, cells have evolved certain types of DNA damage repair (DDR) mechanism. BER is used to repair SSBs and HR, NHEJ, MMEJ, and SSA are used to repair DSBs. If repaired correctly, the cell will remain a healthy cell. However, if repaired incorrectly, these cells will undergo senescence and apoptosis, leading to aging, disease, and cancer.

Many signaling pathways and over 450 related proteins are involved in DDR processes (Jackson and Bartek, 2009; Pearl et al., 2015; Mauri et al., 2020). In addition, different DNA repair mechanisms are used to repair different types of damage, such as homologous recombination repair, nonhomologous end joining (NHEJ), base excision repair (BER), nucleotide excision repair, and mismatch repair (Huang and Zhou, 2020). These repair methods and the outcomes of DNA damage are shown in Figure 1. DDR processes are important for protecting against tumorigenesis. Defects in gene expression and genomic instability are associated with a high risk of gastric cancer and significantly contribute to tumorigenesis and gastric cancer development. The survival of DDR patients with a defective DDR can be improved by the development of therapeutics targeting the DDR.

Many genes also play an important role in the process of DDR, such as 53BP1, BRCA1, RAD51, ATM, and ATR. 53BP1 is a key mediator involved in DSB repair, maintaining the balance between repair pathway selection and genome stability. Recent evidence suggests a molecular mechanism that encodes 53BP1 and DNA break response effectors to DSB sites and promotes NHEJ-mediated DSB repair through 53BP1 (Lei et al., 2022). The roles of the BRCA1 and RAD51 genes are mainly to maintain genome integrity through different mechanisms in response to DNA damage, and its maladjustment is related to the development of a tumor and the change of sensitivity to chemotherapy drugs. Studies have shown that high cytoplasmic expression of BRCA1 has a higher overall survival (OS) rate in gastric cancer, whereas nuclear expression of BRCA1 usually indicates adverse outcomes (Wang et al., 2018). Studies also have shown that gastric cancer tissues have higher levels of RAD51 expression compared with normal tissues (Xu et al., 2021). Therefore, BRCA1 and RAD51 can be used as biomarkers for the clinical diagnosis of gastric cancer to evaluate the prognostic effect of the disease. ATM is involved in DDR through downstream interactions with BRCA1 and other proteins involved in DSB repair. DNA damage-induced ATM activation promotes β-TRCP-mediated ubiquitination and destruction of ARID1A in gastric cancer cells, thereby exacerbating the development of gastric cancer (Jiang et al., 2019). Moreover, novel studies revealed that germline pathogenic variants in ATM genes are associated with a high and moderate risk of many cancers (Hall et al., 2021). ATR is considered an important direction in cancer therapy because of its deleterious effects on cancer cells that contain defects in homologous recombination. Recent studies have shown that AZD6738, as a novel oral ATR inhibitor, can induce synthetic death of gastric cancer cells through ATM defect (Min et al., 2017; Sheridan, 2018). Understanding the role and association of these genes in DDR will facilitate further clinical research to develop new treatment strategies for gastric cancer. Some genes/proteins that function in DDR in gastric cancer are shown in Table 1.

**TABLE 1:** Genes/proteins involved in DDR in gastric cancer cells.

Gene/Protein	Function in DDR	Prognosis	References
BRCA1	A crucial component of HR pathways in DSB repair	Nuclear expression predicted poor outcomes, but high expression of cytoplasmic BRCA1 had a significantly favorable overall survival	Hee Sung et al., 2019; BRCA
BRCA2	A vital component of HR pathways in DSB repair	High expression of cytoplasmic BRCA1 had a significantly favorable overall survival	BRCA; Joel Del Bel et al., 2022
EXO1	Involved in the HR pathway of DSB repair and SSB repair	NO	Lifeng et al. (2012)
KU70/KU80	Ku protein binds to DNA DSB ends and plays a crucial part in NHEJ	An abnormal expression may promote the occurrence of gastric cancer	Wei et al. (2013)
ATM	Involved in DSB repair and activates the DNA damage checkpoint	ATM expression with MIS can be regarded as a prognostic marker	Jin Won et al., 2013; Hannes et al., 2015; Yali et al., 2020
XRCC1	A crucial part of BER for SSBs	Expression of XRCC1 can be regarded as a prognostic marker of gastric cancer recurrence	Bushra et al., 2018; Wang et al., 2012
TP53	Induces cell cycle arrest and apoptosis and blocks DNA repair	TP53 mutations inhibit tumor immunity in gastric cancer	Zehang et al. (2018)
DNA-pkcs	A crucial part of the NHEJ pathway of DSB repair	An abnormal expression may promote the occurrence of gastric cancer	Fu-Rong et al. (2017)
RPA	A crucial part of the HR pathway of DSB repair	RPA may serve as a biomarker or therapeutic target to improve the prognosis of patients with gastric cancer	Efi et al., 2020; Yujie and Chaoran, 2020
RAD51	A crucial part of the HR pathway of DSB repair	RAD51 expression can occur and is regarded as a valuable prognostic marker	Huiying et al. (2021)

In addition, the cell cycle checkpoint genes Wee1, CHK1, and CDK also play irreplaceable roles. The CDK family plays a key role in regulating multiple signaling pathways concerning transcription and cell cycle processes. CDK affects DNA repair and contributes to the fidelity of cell division and the maintenance of genomic integrity after DNA damage (Kciuk et al., 2022). It has been shown that reduced Wee1 activity leads to ectopic activation of CDK1 activity, which drives unrepaired DNA into mitosis prematurely, leading to mutations (Bukhari et al., 2022). Therefore, Wee1 inhibitors can also be used to treat different types of cancer and regulate therapeutic immune responses. CHK1 is a key regulator of the cell cycle in DDR. CHK1 plays an important role in promoting the survival and growth of gastric cancer cells, which is an effective therapeutic target for gastric cancer. Recent clinical trials have shown that CHK1 inhibitor LY2606368 can induce DNA damage and inhibit cancer proliferation (Koustas et al., 2020). However, other researchers have speculated that CHK1 inhibitors may also lead to CHK1 inhibitor toxicity by increasing DNA damage in nontumor cells (Brooks et al., 2021). If some inhibitor compounds acting on a DDR can be found to achieve the targeting therapy, it will pave the way for the treatment of gastric cancer and improve the survival time and the prognosis of patients.

## DNA Damage Repair in Gastric Cancer Because of *Helicobacter pylori* Infection and Other Risk Factors

Risk factors for gastric cancer include many nonmodifiable variables, such as age, sex, and race. Other risk factors are controllable, such as infection with *Helicobacter pylori*, smoking, and a diet high in nitrites and nitrates (Lawrence et al., 2012). There are also several relatively rare risk factors, such as a history of previous stomach surgery and hereditary diffuse gastric cancer (CDH1) (Samantha et al., 2015). Next, we will focus on the mechanism by which *Helicobacter pylori* and other risk factors cause gastric cancer.

Nitrite is a common potential risk factor for gastric cancer. Because the stomach is acidic, nitrite is protonated in the stomach to form nitrous acid. Nitrous acid reacts directly with DNA to form a deaminated base. All of these changes lead to mutations when DNA polymerase is at work replicating DNA (Zhang et al., 2019). Nitrite can also react with food to form N-nitrosamines, some of which are carcinogens to humans because they can react with DNA as alkylation reagents to produce admixtures that can cause harm. At the same time, irregular diet, environmental factors, drinking, and smoking can also lead to tumorigenesis and progression of gastric cancer (Praud et al., 2018).

*Helicobacter pylori* is a strongly virulent Gram-negative bacterium that colonizes the stomach of almost half of all people and is classified as a class Ⅰ carcinogen of gastric cancer (Helicobacter pylori Infection, 2022). *Helicobacter pylori* is a dominant hazard factor for the occurrence mechanism of gastric cancer and is responsible for approximately 90% of new cases. *Helicobacter pylori* can induce DNA damage that can lead to genomic instability and ultimately result in the formation of gastric cancer (Sokolova and Naumann, 2019b). People infected with the bacterium frequently suffer chronic nonatrophic gastritis, eventually resulting in gastric cancer through a complex series of changes. The way in which *Helicobacter pylori* causes DNA damage and then results in gastric cancer has always been a hot topic of research. Next, we summarized the interaction between *Helicobacter pylori* and DNA damage and the effects of this damage on the development of gastric cancer.

Certain cancers in the body develop as a result of the direct carcinogenic effects of certain tumorigenic substances or as a result of genomic instability caused by accompanying inflammation and DNA damage (Kim et al., 2019b). *Helicobacter pylori* infection can induce both innate and adaptive immune responses, including oxidative stress, which leads to DNA damage (Reyes and Peniche, 2019). The build-up of DNA damage can eventually lead to mutations that activate or inactivate tumor suppressor genes. Oxidative stress leads to the production of ROS/RON, and their concentration determines their effect on the body (Han et al., 2022). ROS/RON-mediated DNA damage leads to the breakage of chemical bonds, which is the most common mechanism of carcinogenesis after *Helicobacter pylori* infection. Once the body is infected by *Helicobacter pylori*, innate and acquired immune responses occur, and then an interrelated cellular response produces ROS/RON, leading to oxidative stress. According to previous studies, APE-1 is the main regulator of the cellular response to oxidative stress. APE-1 can repair the sites of oxidative damage in DNA, reduce the activity reduction of many transcription factors, decrease the damage because of ROS and RON in cells and tissues, and maintain the mitochondrial function (Futagami et al., 2008). APE-1 is involved in the transcriptional regulation of genes involved in the adaptive response to oxidative stress and the BER pathway.

**FIGURE 2:**
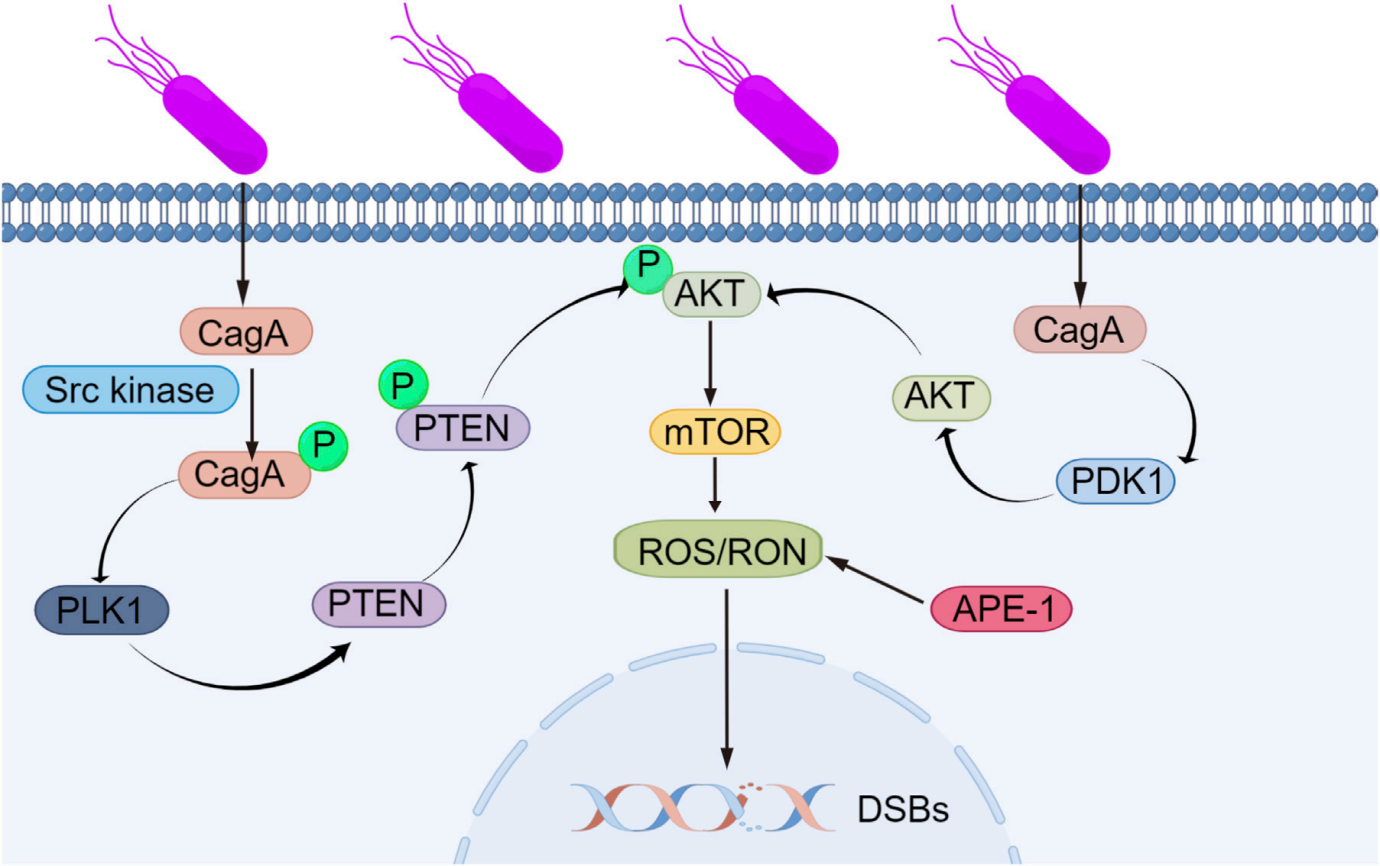
Mechanism by which *Helicobacter pylori* induces DNA damage, leading to genomic instability. *Helicobacter pylori* can express the virulence factor CagA, which is recognized by cells and phosphorylated via Src family kinases. Phosphorylated CagA affects the expression of PLK1, leading to the phosphorylation of PTEN and AKT, while unphosphorylated CagA interacts with PDK1. The activation of AKT kinase leads to subsequent activation of the mTOR complex, producing ROS/RON, and the accumulation of RON/RON in cells causes DNA damage and genomic instability, leading to gastric cancer.

ROS and RON are also involved in many related pathways in the body. The PLK1/P13K/Akt pathway plays an important role in the development of gastric cancer and is inseparably related to ROS and RON. Next, we will introduce the mechanisms of the PLK1/P13K/Akt pathway in detail. *Helicobacter pylori* can express CagA, a virulence factor that is recognized by cells and phosphorylated by Src family kinases (Zhu et al., 2016). CagA possesses functions that are distinct from conventional toxins and can counterbalance the activity of the established *Helicobacter pylori* toxin VacA. Meanwhile, phosphorylated CagA can regulate the expression of PLK1 and then phosphorylate PTEN and AKT, while nonphosphorylated CagA can regulate PDK1 to some extent (Su et al., 2022). The activation of AKT kinase leads to the activation of the mTOR complex, producing ROS/RON, and the accumulation of RON/RON in cells leads to DNA damage and genomic instability, leading to gastric cancer. The mechanism by which *Helicobacter pylori* induces DNA damage through the PLK1/P13K/Akt signaling pathway, leading to gene stability and ultimately gastric cancer, is shown in Figure 2.

Research results have shown that long-term *Helicobacter pylori* infection interferes with the activity of the electron transport chain and damages oxidative phosphorylation, resulting in changes in APE-1 gene expression and decreasing APE-1 expression. Furthermore, there is a certain relationship between APE-1 gene polymorphisms and the tumorigenesis and development of gastric cancer. Chronic *Helicobacter pylori* infection may inhibit APE-1 expression and ultimately lead to genomic instability (Chattopadhyay et al., 2010). Genomic instability is an evolving feature that is caused by mutations in DNA repair genes to drive the development of cancer (Asatryan and Komarova, 2016). Genomic instability includes microsatellite instability (MSI), chromosomal instability (CIN), and the improper activation of telomerase. Microsatellites are repeats of DNA sequences that are almost randomly distributed in all genomes (Polom et al., 2018a). During the process of replication and recombination, errors such as the insertion or deletion of bases can lead to gene mutations that cause MSI. MSI results in the abnormal expression of target genes and ultimately leads to the tumorigenesis of gastric cancer (Silva-Fernandes et al., 2017). As important evidence, the expression rate of MSI is more obvious in elderly women with gastric cancer (Polom et al., 2018b).

High-frequency mutations in microsatellite regions are an indicator of MSI in the DNA sequence, but if such genomic changes occur at the chromosomal level, it is regarded as CIN (Maleki and Röcken, 2017), and its definition continues to change as different types of cancer are continually studied. Some groups have referred to CIN as aneuploidy or polyploidy, while others have defined CIN as multiple structural rearrangements or frequent changes in the chromosome number (Tsai et al., 2018). The formation of CIN is caused by the breakdown of the DNA replication fork induced by an oncogene, which leads to DSB and genomic instability (Kohlruss et al., 2021). Although CIN in cancer has been studied extensively, its exact cause remains unclear. To reveal the exact etiology of CIN in tumors, further studies are needed to investigate its mechanism and the affected pathways.

**FIGURE 3:**
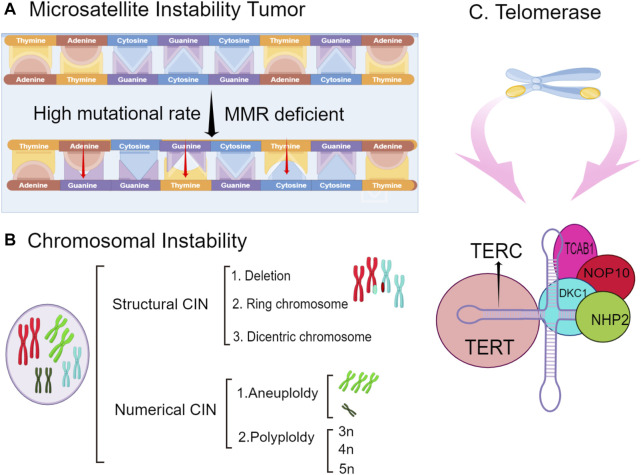
The types of genomic instability include microsatellite instability (MSI), chromosomal instability, and telomerase inactivation. **(A)**. tumorigenesis because of MSI is caused by the abnormal regulation of the expression of target genes **(B)**. chromosomes undergo structural or numerical changes, such as deletions and exchanges and aneuploidy and polyploidy, respectively. **(C)**. telomerase inactivation on chromosomes can lead to genomic instability and gastric cancer. The structure of telomerase is shown.

Telomerase is a reverse transcriptase that uses its own DNA as a template to synthesize telomeres to supplement the telomeres lost during the process of cell division and proliferation so that cells can continue to divide and proliferate (Yucheng and Amir, 2016). Telomerase is an indispensable factor in tumor immortalization and tumorigenesis (Hiyama et al., 1995). According to clinical research data, the telomerase activity in chronic atrophic gastritis cells caused by *Helicobacter pylori* infection is much higher than that in normal cells. The decreased length of telomerase is also a contributing factor to the poor prognosis of gastric cancer (Hiyama et al., 1995). A shortened telomere length will be recognized by the body as a DSB, thereby activating the DNA damage reaction pathway to protect the body, but unfortunately, this aberrant activation can cause great harm. The telomere length has important research value in studies on neurobiology and gastrointestinal microbes. Genomic instability leads to the loss of tumor suppressor genes and the improper activation of oncogenes, triggering uncontrolled cell proliferation and continued malignant cell development (Haojian et al., 2021). This mechanism of gene instability is shown in Figure 3.

In conclusion, we also review our previous work on the relationship between *Helicobacter pylori* and DNA damage. We have confirmed that transforming growth factor-β is an important mediator in the pathogenesis of *Helicobacter pylori* (Nianshuang et al., 2016). We have identified the molecular mechanisms underlying the interaction between the Hippo and Wnt signaling pathways and elucidated their roles in tumorigenesis of the gastrointestinal tract, especially the intestine, stomach, and liver (Li et al., 2019). We further verified that the inhibition of autophagy activates the DNA damage response and initiates gastric tumorigenesis via Rad51 ubiquitination in response to *Helicobacter pylori* infection (Chuan et al., 2020). In the future, we will continue to study the strong link between *Helicobacter pylori* and gastric cancer.

## Therapeutic Approaches for Treating DNA Damage in Gastric Cancer

Considering that the individual DDR capacity of gastric cancer patients varies greatly, the diversity of DDR genes not only is a crucial genetic element but also provides a novel approach to treat gastric cancer and will become a focus and hotspot in the cancer research field. Regarding the treatment of gastric cancer, some approaches have been proposed and validated. For example, the US Food and Drug Administration (FDA) has approved some special DNA-damaging agents for the clinical treatment of gastric cancer, such as PARP inhibitors. Furthermore, certain chemotherapy and radiotherapy regimens are also effective. Below is a summary of the DNA-damaging agents and chemoradiotherapy used in the current treatment of gastric cancer.

## DNA-Damaging Agents in Gastric Cancer

DNA-damaging agents induce diverse types of DNA damage, mainly DSBs and SSBs. These types of DNA damage are sensed and repaired by proteins involved in the DNA damage response. Therefore, the abnormal expression of a particular DNA damage response protein could be a biomarker of resistance or of a favorable response to therapies that induce the corresponding types of DNA damage. For instance, SLFN11 has been shown to suppress gastric cancer growth both *in vitro* and *in vivo* and to enhance the capacity of cisplatin to induce S-phrase arrest and apoptosis in gastric cancer. Therefore, the use of SLFN11 has contributed to improvement in the prognosis and survival of cancer patients (Yaojun et al., 2019).

Aurora kinase A (AURKA) is highly overexpressed in gastric cancer and inversely correlated with prognosis. AURKA restricts survivin ubiquitylation and degradation in gastric cancer to promote drug resistance, and hence, the AURKA–survivin axis can be targeted to enhance the efficacy of DNA-damaging agents in treating gastric cancer (Kamran et al., 2017). In recent years, a retrospective multicenter data analysis of gastric cancer with BRCA1 or BRCA2 germline mutations (gBRCAm) was conducted to identify 10 gastric cancer patients with gBRACm, 6 of whom had metastatic disease. The results of the analysis demonstrated that the median OS was 4.5 months for all 10 gastric cancer patients, 55.5 months for patients with operable disease, and 32 months for patients with metastatic disease. These preliminary data suggested that gBRCAm is associated with favorable outcomes in gastric cancer patients (Naama et al., 2020). However, the data samples of these cases are only 10, and further experimental research studies are needed to verify them. In contrast, a large number of DNA-damaging agents have been developed, and among them, some have partly been tested for their ability to enhance DNA damage-induced tumor cell killing in preclinical studies and clinical trials. As this technology improves, more uncertainties will be resolved in future experiments.

Different types of DNA damage trigger phosphorylation-mediated signaling cascades that lead to stimulating specific cellular responses. In the NF-KB signaling pathway, the transcription factor RelA is critical to these DNA damage response pathways. Different DNA damage agents can induce different cell outcomes through transcription factor RelA, but its specific coordinated signal transduction mechanism remains unclear (Campbell et al., 2021). The P13k/AKT signaling pathway also plays an important role in DDR. The dual inhibition of P13k/AKT signaling and DNA damage checkpoints in p53-deficient cells accelerates rapid apoptotic cell death during the G (2) period (Skladanowski et al., 2007). Maintenance of genomic integrity after DNA damage depends on the activation of the tumor suppressor P53, which then coordinates the DNA repair system/cell cycle checkpoint. The Wnt/β-catenin signaling pathway is one of the main targets of p53. Therefore, when related DNA damage agents act on the Wnt/β-catenin signaling pathway, DNA damage will occur, leading to genomic instability and thus aggravating the tumorigenesis and progression of gastric cancer (Karimaian et al., 2017). It is worth adding that DNA damage agents can also activate p53 through different upper signaling pathways, such as SAPK signaling (Shi et al., 2021). When the mechanism of the signal pathway involved in the occurrence and development of gastric cancer is clearly studied, its treatment will also become feasible.

Some related inhibitors are also being developed, such as Wee1 inhibitors, CHK1 inhibitors, and PARP inhibitors. Wee1 inhibitors are undergoing clinical trials. The Wee1 inhibitor AZD1775 has an effective checkpoint inhibitory activation effect, which can significantly inhibit the proliferation of gastric cancer cells and induce apoptosis and cell cycle arrest, especially in gastric cancer cells with high Wee1 expression (Chen et al., 2018). When combined with CHK1 inhibitors, Wee1 inhibitors can overcome the resistance of tumor cells to CHK1 and thus enhance the anticancer activity (Li et al., 2020). In the meantime, the combination of CHK1 inhibitor LY2606368 and PARP inhibitor BMN673 showed a better synergistic anticancer effect (Parmar et al., 2019). It can be seen from these studies that Wee1, CHK1, PARP, and others are all valuable targets in the treatment of gastric cancer, and the combination of different target-related inhibitors may be a more effective strategy for the treatment of gastric cancer in the future, which improves the overall treatment outcome in patients with advanced gastric cancer.

For now, PARP inhibitors have made great strides in the treatment of cancer. We also systematically analyzed the application of PARP inhibitors in gastric cancer. ADP-ribosylation is the modification of target proteins by ADP-ribosyltransferase using NAD+ and ADP-ribosyltransferase (Hopp et al., 2019), which can alter the physical and chemical properties of target proteins in many important processes (Lin and Caroll, 2018), such as DNA repair, transcription, telomere length and senescence, protein degradation, apoptosis, and necrosis (Veneris et al., 2020). The only known ADP-ribosylated proteins are the members of the PARP family. This family of proteins consists of 17 members with different domains, activities, subcellular localizations, and functions. PARP is a DNA sensor; its polymeric ADP–ribose strands act as a platform for protein signaling to coordinate the DNA repair process (D'Andrea, 2018). However, PARP also affects the degree of DNA damage. For lower degrees of DNA damage, PARP activity can be beneficial for DNA repair and cell survival, but under ischemic conditions or in the presence of severe inflammation, widespread DNA damage can occur, activating at least two different mechanisms to cause cell death, namely, cell necrosis induced by energy depletion and apoptosis-inducing factor–dependent apoptosis (Luo and Kraus, 2012). It is also worth noting that some studies had found that PARP can also affect many important biological activities in cells by changing the AMP/ATP ratio (Min and Im, 2020).

Next, we will focus on the main mechanisms by which PARP inhibitors combat tumors in cancer cells. First, we can think of PARP inhibitors as “poisons” that cause the PARP enzyme to become trapped on the DNA strand, facilitating the formation of poly(ADP-ribose) chains from NAD+ (Kim et al., 2021). Second, PARP inhibitors inhibit the repair of NHEJ, leading to cell apoptosis or necrosis after DNA damage occurs (Gupta et al., 2018). Third, some new studies have shown that PARP inhibitors reduce the repair efficiency of MMEJ, an auxiliary DNA repair mechanism (Tomasini et al., 2021). This speeds up the process of apoptosis, or death, in cancer cells that do not initiate the required DNA repair. Several PARP inhibitors have been developed. For example, olaparib is an oral PARP inhibitor used for the treatment of gastric cancer. It can both activate DDR pathways and reactivate DNA checkpoints. In addition, olaparib can be combined with many other substances to enhance the capacity of these repair pathways. A long-standing study showed that when olaparib was combined with paclitaxel, olaparib plus paclitaxel was well tolerated and led to a statistically significant improvement in OS and ATM-pts with a larger benefit in ATM-pts (Yung-Jue et al., 2013). In recent years, some scholars have pointed out that the combination of the P13K inhibitor BKM120 with olaparib can inhibit the proliferation of gastric cancer cells with ARID1A deficiency (Lin et al., 2018). After this, olaparib plus AZD1775 was found to augment the antitumor activity by disrupting DDR pathways and DNA damage checkpoints (Xiaoting et al., 2018). Another study demonstrated that olaparib combined with talaporfin photodynamic therapy can improve the efficacy by inducing the formation of PARP-DNA complexes in gastric cancer (Mamoru et al., 2021). This strategy may be potentially useful for the treatment of gastric cancer. Although olaparib and other substances have been used clinically, the pathogenesis of gastric cancer is different among different patients. Therefore, olaparib still needs further development (National Library of Medicine, 2006). In Table 2, we describe the clinical and nonclinical uses of five typical PARP inhibitor drugs. PARP inhibitors are particularly sensitive and fit with the new concept of “synthetic lethality,” in which the function of two repair pathways is synergistically lost, leading to cell death (Ashworth and Lord, 2018). Therefore, the clinical success of PARP inhibitors has brought new hope for synthetic lethal anticancer therapy, representing one of the next generations of anticancer drugs targeting DDR. In essence, PARP inhibitors prevent the repair of single-stranded DNA breaks, leading to cell apoptosis.

**TABLE 2:** A summary of five PARP inhibitors in clinical and preclinical development.

Name	Research Stage	Target	Efficacy Against Gastric Cancer	References
Olaparib	FDA approval (OC)	PARP1 and PARP2	Activates the HR pathway and repairs the DNA checkpoint	Sylvia et al. (2018); Xiaoting et al. (2018)
Niraparib	FDA approval (OC)	PARP1 and PARP2	Unclear at the moment (ongoing study)	Kathleen et al. (2019)
Veliparib	Phase Ⅲ	PARP1 and PARP2	Targets PARP-mediated DNA damage response pathways	Vinod Vijay et al. (2016)
Talazoparib	FDA approval (BC)	PARP1 and PARP2	Mostly used in breast and ovarian cancers, but not in gastric cancer	Dong et al., 2021
Rucaparib	FDA approval (OC)	PARP1 and PARP2	Used in ovarian cancer, but not in gastric cancer	Yahiya (2017)

In the case of circumscribed gastric cancer, surgical resection is the most suitable treatment strategy (Riquelme et al., 2015). However, most gastric cancers are diagnosed as advanced and incurable when they are discovered, which limits traditional treatments and makes the development of effective anti-DDR therapeutic strategies so promising. Anti-DDR therapeutic strategies have obvious advantages over other therapeutic strategies. A recent comparative clinical study showed that the experimental group receiving anti-DDR therapeutic strategies had longer OS, reduced tumor response rate and serious adverse reactions, and improved prognosis and quality of life (Reddavid et al., 2021). Anti-DDR therapeutic strategies are the most advanced and can achieve precise effects similar to the “guiding rocket,” which directly acts on the cancerous parts and tissues, causing less damage to normal cells and resulting in better therapeutic effects (Patel and Cecchini, 2020). The development of anti-DDR therapeutic strategy-related pathways and protein inhibitors will help humans to achieve personalized treatment for patients with advanced gastric cancer.

## Efficient Management of Gastric Cancer via Chemoradiotherapy

As the standard treatment for cancer, chemoradiotherapy has been extensively applied to clinical practice worldwide; however, its efficacy in the eradication of cancer cells, suppression of metastasis, and improvement of the OS of patients is poor. The mechanism of chemoradiotherapy primarily induces DNA damage to kill cancer cells; thereby, the efficacy of chemoradiotherapy depends on the generation of DNA damage. After long-term exposure to endogenous and exogenous DNA damage, the body may evolve a DNA damage response mechanism. It is normally used to sense and repair DNA damage. If the damaged lesion is completely repaired, the cell will survive; otherwise, the cells will die.

Compared with open gastrectomy, laparoscopic surgery in patients with advanced gastric cancer following neoadjuvant chemotherapy causes fewer complications and leads to a better prognosis, faster recovery of intestinal function, and prolonged survival (Liao et al., 2021). Selected gastric cancer patients with limited regional lymph node recurrence may benefit from radiotherapy combined with chemotherapy and high-dose radiotherapy (≥54 Gy) leads to a better progression-free survival and tends to extend the OS (Liang et al., 2020). A recent analysis of related research data demonstrated that tumor bleeding could be adequately controlled by palliative radiotherapy in patients with unresectable advanced gastric cancer (Jesang et al., 2021). Receptor tyrosine kinase MET overexpression is frequently observed in a range of different cancers, is associated with poor prognosis, and has been investigated in several clinical trials (Georgina et al., 2021). Olaparib can be used as an adjunct to chemotherapy to more effectively treat gastric cancer. It activates DDR and DNA checkpoint pathways to achieve a better treatment effect in gastric cancer. Some adjuvants in combination with radiotherapy may have adequate benefits in other areas as well (Marie et al., 2017). Pembrolizumab synergizes with radiotherapy, resulting in significantly improved outcomes in patients with nonsmall-cell lung cancer (NSCLC). Data from the KEYNOTE-189 phase III trial confirm that adding pembrolizumab to first-line chemotherapy improves the outcomes of patients with metastatic NSCLC, improving survival by several months (Sidaway, 2018).

Although chemotherapy and radiotherapy have not worked as well as we would like, more trials are needed to yield further innovations in cancer treatment.

## Conclusion and Perspectives

Abundant data have proven that DDR pathways play a crucial role in the tumorigenesis, progression, treatment, prognosis, and other aspects of breast cancer. Hence, for the treatment of gastric cancer, precise DDR mechanisms in cells should be clarified with further experimentation. In this article, we reviewed latent DDR mechanisms and the DDR in gastric cancer associated with *Helicobacter pylori* infection, which represent novel therapeutic targets. In general, the road ahead is long, and further research will be indispensable. Further studies on DDR mechanisms will aid in providing a more comprehensive understanding of the etiology of gastric cancer (and other tumors) and will provide evidence regarding the best therapies for individual patients. Moreover, it is expected that the incidence and mortality of gastric cancer will sharply decrease with the efforts of scientific researchers.
